# It is Not Time to Kick Out Radiologists

**DOI:** 10.1007/s41649-024-00325-1

**Published:** 2024-12-03

**Authors:** Yuta Nakamura, Yuki Sonoda, Yosuke Yamagishi, Tomohiro Kikuchi, Takahiro Nakao, Soichiro Miki, Shouhei Hanaoka, Takeharu Yoshikawa, Osamu Abe

**Affiliations:** 1 Department of Computational Diagnostic Radiology and Preventive Medicine, The University of Tokyo Hospital, Tokyo, Japan; 2 Division of Radiology and Biomedical Engineering, Graduate School of Medicine, The University of Tokyo, Tokyo, Japan; 3 Data Science Center, Jichi Medical University, Shimotsuke, Japan

Muyskens et al. (2025), in their recent original article “When can we Kick (Some) Humans ‘Out of the Loop’? An Examination of the Use of AI in Medical Imaging for Lumbar Spinal Stenosis”, boldly challenged the prevailing notion on medical imaging artificial intelligence (AI). Currently, it is widely thought that AI is a support tool under human supervision, AI must not fully replace humans, and humans remain crucial to healthcare (Alowais et al. 2023). Instead, Muyskens et al. suggested that under certain conditions, some healthcare processes could be entirely AI-driven with no human involvement. They argued that *Spine AI* (Hallinan et al. 2021), which automates preoperative lumbar spine MRI measurements, is equivalent to human radiologists and can fully replace them with little harm (Muyskens et al. 2025).

While we are not bioethics experts, we find this article ambitious and highly stimulating. However, as experts in both radiology and AI, we must raise one critical opposition. The authors have a serious misbelief that AI will soon be able to fully replace human radiologists, which potentially harms patients.

## Radiologists Cannot be Replaced by AI Yet

Radiologists are far more than mere measurement tools, and AI is still in development. Thus, the scenario where human radiologists can be completely kicked out of healthcare for lumbar spinal stenosis (LSS) is not feasible yet. This is not about protecting the radiologists’ pride or jobs, but about preventing potential harm to patients.

Approximately 5% of lumbar spinal MRIs reveal incidental extraspinal findings that are clinically significant (Broadhurst et al. 2023). For example, incidental lung cancer might appear on the edge of an MRI image in the Spine AI case study scenario, too. Then, human radiologists would alert the referring clinician through radiology reports or urgent phone calls (Berland et al. 2010), but Spine AI would do nothing.

Therefore, the only diagnostic process where Spine AI might kick out human radiologists safely is limited to the spine measurement, not the entire evaluation of preoperative lumbar spinal MRIs. It is extremely harmful to try to fully automate image diagnosis with such a single-task AI.

## Radiologist’s Proficiency in Patient Care Loops

At first glance, radiologists might appear only to respond to direct requests. However, radiologists’ work goes far beyond that. Radiologists are trained to, and continue to, thoroughly evaluate everything in any imaging study, in addition to addressing the direct request (Drew et al. 2013).

A lumbar spinal MRI captures not only the lumbar spine but also other body parts. Thus, radiologists check out potential issues like the following: an incidental lung tumor, an abdominal aortic aneurysm, asymptomatic hydronephrosis, or lymphadenopathy. Radiologists might also detect subtle localized pancreatic atrophy suggesting early pancreatic cancer.

Moreover, the spine and spinal cord can develop various abnormalities, such as bone metastases, hematologic disorders, granulomatous diseases like sarcoidosis, and demyelinating diseases like multiple sclerosis (Hanrahan and Shah 2011, Mohajeri Moghaddam and Bhatt 2018).

Patients often have comorbidities that are not always mentioned in the referral but still require attention. Radiologists also assess them by reviewing past imaging studies and medical records. For a patient undergoing chemotherapy for recurrent rectal cancer, radiologists evaluate changes in metastatic lesions. For a patient with a history of autoimmune pancreatitis, radiologists look for pancreatic enlargement suggesting a relapse.

Radiologists thoroughly review various image findings even for preoperative MRI. This comprehensive evaluation often keeps them from missing any clinically significant findings. If Spine AI expels human radiologists, orthopedic surgeons must be in charge of identifying various incidental findings instead. Moreover, delay in detection of incidental findings could not only harm the involved patient, such as in cases of malignancies, but also other patients, as seen with active pulmonary tuberculosis or COVID-19 pneumonia. This responsibility seems excessively burdensome for orthopedic surgeons.

Therefore, the ideal use of Spine AI would be to automate only the spine measurements, while human radiologists should handle the assessment of the other image findings.

## AI is Not Professional Yet

The key difference between current AI and human radiologists is that human radiologists have cross-organ and cross-disciplinary skills, while AI does not. The point here is not that human radiologists use different abilities for different imaging studies, but rather that they employ numerous skills commonly across any imaging studies to prevent patient harm. Therefore, AI must also develop this versatility to be able to replace human radiologists. However, no approaches have successfully realized versatile AI.

The first approach is a bottom-up method to assemble numerous high-performance single-task AI models to work together. The gap between AI and human radiologists is narrowing in diagnosing breast cancer with mammography (Lauritzen et al. 2024). However, with a few exceptions like this, tasks where AI can rival human radiologists are still limited. Many tasks performed by human radiologists remain, and it is draining to develop countless single-task AI models to fill the gaps.

The second approach is a top-down method to use engineering tricks to make AI cover all abnormal findings, such as unsupervised anomaly detection models. They can detect any image finding deviating from the distribution of a healthy population (Hojjati et al. 2024). Apparently, such anomaly detection models might substitute human radiologists’ work, making clinicians only need to consult the relevant department of the body parts where an anomaly is detected. Unfortunately, however, anomaly detection models have a fundamental limitation that they cannot distinguish the type or severity of the detected anomalies (Nakao et al. 2021). This limitation potentially leads to a flood of unnecessary and non-urgent consultations including false positives. Hence, radiologists still outperform AI in triaging abnormal findings.

The third approach is to train extremely large models with vast amounts of data like ChatGPT, hoping that they will exhibit versatility. Although these large models might seem competent in everything, their current performance on image diagnosis is far below expectations. For instance, when GPT-4 V was tested on the Japanese diagnostic radiology board examination, no improvement was observed when images were added to the text inputs (Hirano et al. 2024). Furthermore, when GPT-4 V and Gemini Pro were tested with datasets of radiological images on medical articles, their performance was below random guessing, despite the datasets being public (Yan et al. 2024). These indicate that large models are still far from accurate image diagnosis.

Even with cumulating all currently available AI models, most of the tasks performed by human radiologists on any single imaging study cannot be replaced. At present, therefore, complete replacement of human radiologists with AI is harmful to patients and cannot be justified. Against Muyskens et al.’s opinion, countries short of human radiologists are no exceptions, because teleradiology is a far better choice to meet the demand (Ewing and Holmes 2022).

## AI Needs Long-Time Management

Current AI lacks the versatility necessary to fully replace human radiologists in any single imaging study. Then, in the future, what should we do when a versatile AI model is finally developed, whose performance matches human radiologists across numerous radiological tasks?

Before expelling human radiologists, we strongly urge taking a moment to answer another crucial question: whether the AI’s capabilities are truly sufficient and whether there is a management framework to maintain those capabilities over time.

AI products for long-term operation are completely different from AI research prototypes (Makinen et al. 2021). It is far more difficult to maintain AI’s performance for many years than to make AI perform well only at a specific moment. AI is vulnerable to *dataset shift*, changes in data distribution from the training to operational phases (Subbaswamy and Saria 2020). AI’s initial high performance can drastically drop due to minor changes in the external environment. Therefore, it is vital to ensure the external validity to make AI robust to withstand such changes (Finlayson et al. 2021).

Dataset shift can occur typically between institutions, but also within an institution (Castro et al. 2020). Radiological departments regularly purchase new equipment (European Society of Radiology 2014) and revise imaging protocols (Zhuo and Gullapalli 2006) for quality assurance in imaging. This update makes imaging data strange to the AI and causes long-term dataset shift. In addition, imaging protocols can be modified on a daily basis for a specific patient, such as to reduce noise from frequent body movement (Boland et al. 2014), which can also confuse AI.

Therefore, even if the AI is for use solely within a single institution, it cannot avoid the necessity of validating its external validity. If AI has insufficient external validity, accumulated changes in the external environment may gradually degrade its performance, potentially necessitating the return of human radiologists.

In the machine learning industry, it is well understood that AI should be updated periodically (Sculley et al. 2015), instead of pursuing a perfect static AI. It is essential to monitor dataset shifts and re-train AI to maintain its performance. This comprehensive and continuous management of the AI lifecycle is referred to as machine learning operations (MLOps). Recent insights into MLOps demonstrate that various technicians and technologies must be orchestrated, even only to maintain AI’s performance (Kreuzberger et al. 2023).

In summary, AI’s performance should be discussed and evaluated together with the framework for long-time management, rather than focusing on a certain point of time.

## Radiologist’s Supervision in AI Management Loops and Patient Care Loops

AI is in individual patient care loops. In addition, AI is also part of a larger loop of long-time management, including development, implementation, monitoring, and re-training. Human radiologists will remain indispensable in both loops.

Let us first consider the larger AI management loop. When implementing new AI, it is crucial to examine its performance in the environment of daily practice in the user’s institution. This verification process has several steps: (i) defining the tasks and acceptable performance levels, (ii) creating a benchmark by collecting imaging data in the user’s institution and assigning gold standard labels, and (iii) testing the AI with this benchmark (Rubin 2019). If the AI is required to rival human radiologists, the gold standard labels in step (ii) should also be created by them. Furthermore, this performance verification process should be repeated in response to change in patient populations, equipment update, and imaging protocol revision (Leming et al. 2023). During this process, human radiologists need to re-label the new images to create new benchmarks.

Next, let us focus again on the smaller loops of individual patient care. Radiologists’ role is not limited to benchmark factory workers during the periodic updates of AI. Another radiologists’ important role is to supervise the AI during image diagnosis. Expecting zero errors from AI is unrealistic, because its performance is typically tuned to prioritize either sensitivity or specificity depending on the use case (Rubin 2019). Additionally, the patterns of errors made by AI differ from those by humans (Liu et al. 2022), and there is a risk of AI temporarily malfunctioning due to unexpected input data issues or technical problems (Evans and Snead 2024). Humans must be aware of signs of such AI errors and confidently reject incorrect AI suggestions. To do so, humans must possess diagnostic capabilities close to those of the AI. Therefore, we believe that radiologists are the most suitable to supervise the use of imaging diagnostic AI in the individual patient care.

Radiologists have traditionally served as interpreters, summarizers, communicators, and quality controllers of medical imaging modalities. Similarly, in the future, radiologists will contribute to safer and more efficient healthcare by serving as interpreters, summarizers, communicators, and quality controllers of AI (Najjar 2023).

## Conclusion

Muyskens et al. have overestimated the current capabilities of medical imaging AI and underestimated the role of radiologists, leading to the misbelief that the AI is about to replace human radiologists. As experts in radiology and AI, we inform the bioethics community that (i) current AI cannot rival human radiologists, who always comprehensively evaluate images, and (ii) the role of human radiologists extends beyond image diagnosis to the management of AI. These perspectives have not been considered in Muyskens et al.’s interdisciplinary discussions on the conditions under which AI might fully replace human radiologists. Therefore, it is not time to “kick out” human radiologists.

## Data Availability

Data availability is not applicable as no datasets were generated or analyzed for this manuscript.

## Declarations

### Declaration on the Use of AI in the Writing Process

All authors declare that generative artificial intelligence (AI) technologies were used only to improve readability and refine language under strict human supervision. No AI-assisted technologies were used to generate content or ideas. All authors carefully reviewed and edited the manuscript to make it accurate and coherent.
